# The Epoxygenases CYP2J2 Activates the Nuclear Receptor PPARα *In Vitro* and *In Vivo*

**DOI:** 10.1371/journal.pone.0007421

**Published:** 2009-10-12

**Authors:** Jessica A. Wray, Mary C. Sugden, Darryl C. Zeldin, Gemma K. Greenwood, Salma Samsuddin, Laura Miller-Degraff, J. Alyce Bradbury, Mark J. Holness, Timothy D. Warner, David Bishop-Bailey

**Affiliations:** 1 Translational Medicine and Therapeutics, William Harvey Research Institute; 2 Centre for Diabetes and Metabolic Medicine, Bart's and the London, Queen Mary University of London, London, United Kingdom; 3 Division of Intramural Research, NIEHS/NIH, Research Triangle Park, North Carolina, United States of America; AgroParisTech, France

## Abstract

**Background:**

Peroxisome proliferator-activated receptors (PPARs) are a family of three (PPARα, -β/δ, and -γ) nuclear receptors. In particular, PPARα is involved in regulation of fatty acid metabolism, cell growth and inflammation. PPARα mediates the cardiac fasting response, increasing fatty acid metabolism, decreasing glucose utilisation, and is the target for the fibrate lipid-lowering class of drugs. However, little is known regarding the endogenous generation of PPAR ligands. CYP2J2 is a lipid metabolising cytochrome P450, which produces anti-inflammatory mediators, and is considered the major epoxygenase in the human heart.

**Methodology/Principal Findings:**

Expression of CYP2J2 *in vitro* results in an activation of PPAR responses with a particular preference for PPARα. The CYP2J2 products 8,9- and 11-12-EET also activate PPARα. In vitro, PPARα activation by its selective ligand induces the PPARα target gene pyruvate dehydrogenase kinase (PDK)4 in cardiac tissue. I*n vivo*, in cardiac-specific CYP2J2 transgenic mice, fasting selectively augments the expression of PDK4.

**Conclusions/Significance:**

Our results establish that CYP2J2 produces PPARα ligands *in vitro* and *in vivo*, and suggests that lipid metabolising CYPs are prime candidates for the integration of global lipid changes to transcriptional signalling events.

## Introduction

Exogenous PPAR activators include a number of fatty acids as well as a variety of eicosanoid, HETEs, HODEs, prostaglandins, and leukotrienes. A number of lipid-metabolising pathways have therefore been suggested as sources of PPAR ligands, however none really fully satisfy the criteria required for them to be regarded as ubiquitous endogenous PPAR ligand generators [1], [2]. The cyclooxygenase, and 5-, 12/15-lipoxygenase pathways are good examples: prostanoid synthase enzymes and lipoxygenase isoforms have a highly tissue-specific expression pattern that do not fully match those of the PPARs and the effects of prostanoid/lipoxygenase enzyme inhibitors or the phenotypes of the corresponding knockout animal do not match those of the PPARs [1]. Phospholipases [3] or lipoprotein lipase [4] can produce PPARα ligands from circulating lipoproteins. However, it is unclear whether these enzymes produce PPAR ligands universally. A very attractive hypothesis is that cytochrome P450 enzymes (CYPs) could provide the link. Similar to related eicosanoids, 8,9-, 11,12-, and 14,15-EET and their CYP4A hydroxylase metabolites can bind and activate a PPARα reporter gene [5], and 8,9-, 11,12- and 14,15-EETs can functionally activate both PPARα [6] and PPARγ [7], [8] *in vitro*. It is not known however, which CYPs act as potential sources of the EETs, or whether CYPs or EETs mediate any functional effects on PPARs *in vivo*.

There are more than 500 CYP genes primarily associated with the metabolism and detoxification of foreign chemicals. A number of CYPs also catalyze the metabolism of lipids by epoxygenases lipoxygenase-like, and ω- and ω-1-hydroxylase activities [9]. The CYP2 gene family of epoxygenases has approximately 25 members. CYP2J2 is the only CYP2J family member expressed in man, and it is localised in the heart and vasculature, throughout the gastro-intestinal and respiratory tracts and in the kidney [9], [10], where it catalyses the conversion of arachidonic acid via the epoxygenase pathway to anti-inflammatory and vascular-protective EETs [10]. Here we show CYP2J2 activates PPARα *in vitro* and *in vivo*.

## Results

### CYP2J2 activates PPARs *in vitro* in an autocrine manner

**Figure 1:**
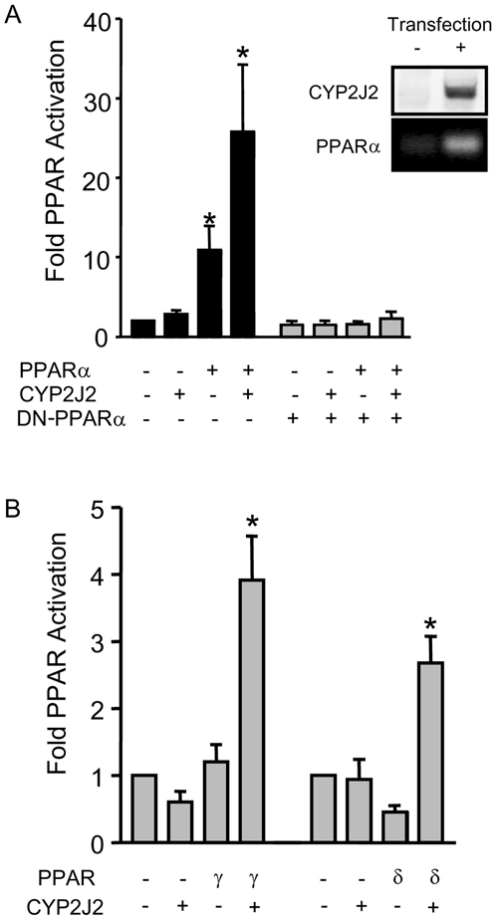
CYP2J2 activates PPAR responses *in vitro*. CYP2J2 synergises with PPARα (A), PPARβ/δ or PPARγ (B) to induce PPAR reporter gene activation. Dominant-negative (DN-)PPARα co-transfected into cells with CYP2J2 and PPARα abolished the ability of CYP2J2 to activate PPARα (A). HEK293 cells were transfected with PPAR reporter genes (pACO.gLuc for PPARα and –γ, and pDR-1 for PPARδ), and pcDNA-CYP2J2, pCMX-PPARα, pCMX-PPARδ, or pCMX-PPARγ alone, or co-transfected with CYP2J2 and the individual PPAR (2J2+α, 2J2+δ, and 2J2+γ). All PPAR reporter gene activation studies are represented as fold luciferase from PPAR response element transfection alone (control), normalised to total protein at 20 h post-transfection. Total plasmid DNA for transfections was normalised using pcDNA3.1 throughout. Data represents n = 9−12 replications from 4 separate experiments. * denotes p<0.05 by one-sample t-test between control and transfected cells. Inset (A) is Western blot for CYP2J2 and RT-PCR for PPARα in cells with either mock transfected (−; pcDNA3.1) or cells transfected with pcDNA-CYP2J2 and pcDNA-mPPARα (+).

**Figure 2:**
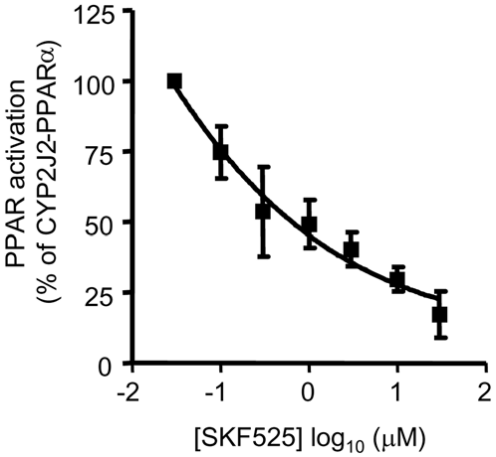
Synergistic activation of PPARα by CYP2J2 requires an active CYP2J2. Cells were co-transfected with PPAR reporter gene, CYP2J2 and PPARα, and treated with the epoxygenase inhibitor SKF525A (0–30 µM). SKF525A caused a concentration-dependant inhibition of PPAR reporter gene activation. Data represents n = 9−12 replications from 4 separate experiments.

Transient transfection of the CYP2J2 cDNA in HEK293 cells produced significant expression of CYP2J2 protein (Figure 1A). The combination of CYP2J2 with PPARα (Figure 1A), PPARδ or PPARγ (Figure 1B) induced a synergistic activation of PPAR reporter genes, with a marked preference in terms of absolute activity for PPARα activation (Figure 1A). pDR-1 was used as a reporter gene for PPARδ activation due to the reported lack of efficacy for pACO on PPARδ responses [11]. A functional PPAR was required for this activation, as no significant reporter gene activation was seen in cells co-transfected with vector reporter gene lacking the PPRE (data not shown), or when cells were co-transfected with dominant-negative (DN)-PPARα [12; Figure 1A). Similarly, the activation of PPAR reporter gene by co-transfection of PPARα and CYP2J2 required *active* CYP2J2, as the epoxygenase inhibitor SKF525A caused a concentration-dependent inhibition of PPARα-CYP2J2 induced PPAR reporter gene activation (Figure 2). These endogenous products of CYP2J2 act in an intracellular manner, as only when cells are co-transfected so that PPARα and CYP2J2 are co-expressed together in the same cell is a significant synergistic activation of the PPAR reporter gene detected (data not shown).

### CYP2J2 activates PPARα and inhibits NFκB activation

**Figure 3:**
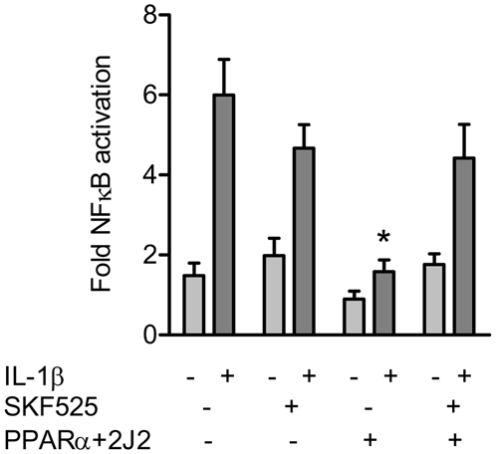
CYP2J2 activates PPARα to inhibit NFκB activation. IL-1β (10 ng/ml; 20 h) induced NFκB reporter gene activation in HEK293 cells. Cells co-transfected with CYP2J2 and PPARα abolished IL-1β-induced NFκB activation. Co-incubation of cells with the epoxygenases inhibitor SKF525A (10 µM) had no effect on IL-1β induced NFκB activation, but reversed the ability of PPARα and CYP2J2 to inhibit IL-1β induced NFκB activation. HEK293 cells were transfected with NFκB reporter gene and pcDNA-CYP2J2 and pCMX-PPARα. Data is represented as fold luciferase from NFκB reporter transfection alone (control), normalised to total protein at 20 h post-transfection. Total plasmid DNA for transfections was normalised using pcDNA3.1 throughout. Data represents n = 6 replications from 3 separate experiments. * denotes p<0.05 by paired t-test between IL-1b and treatments.

PPARα activation inhibits the activation of the pro-inflammatory and survival transcription factor NFκB [1], [2]. IL-1β (10 ng/ml) induced NFκB reporter gene activation in HEK293 cells transfected with control plasmid cDNA. In cells transfected with the combination of CYP2J2 and PPARα, IL-1β induced NFkB activation was completely abolished (Figure 3). Inhibiting CYP2J2 with SKF525A (10 µM) restored the ability of IL-1β to activate NFκB in PPARα and CYP2J2 transfected cells (Figure 3)

### EETs activate PPARα

**Figure 4:**
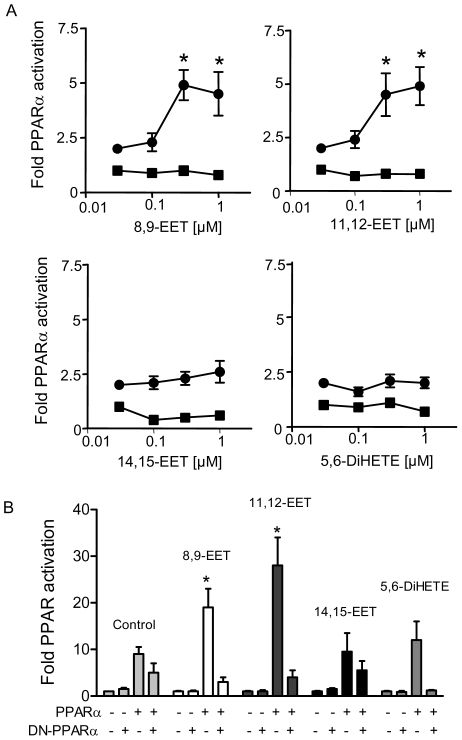
CYP products activate PPARα. (A) 8,9-EET, and 11,12-EET, but not 14,15-EET, or 5,6-DiHETE, induce PPAR reporter gene activation in cells transfected with PPARα (with out PPARα, closed squares; +PPARα closed circles). HEK293 cells were transfected with PPAR reporter gene in the presence or absence of PPARα. 20 h post transfection cells were treated with CYP2J2 products (0–10 µM), for a further 20 h. * denotes p<0.05 by one-way ANOVA followed by Bonferroni's post test. (B) 8,9-EET and 11,12-EET activation of PPARα responses were inhibited by co-transfection with dominant-negative DN-PPARα. Cells were transfected with a PPAR reporter alone or with PPARα, in the presence or absence of DN-PPARα. Cells were treated with CYP2J2 products as indicated (all at 1 µM). Data represents n = 9−12 replications from 3–4 separate experiments. * denotes p<0.05 by one-sample t-test between control and transfected cells.

**Figure 5:**
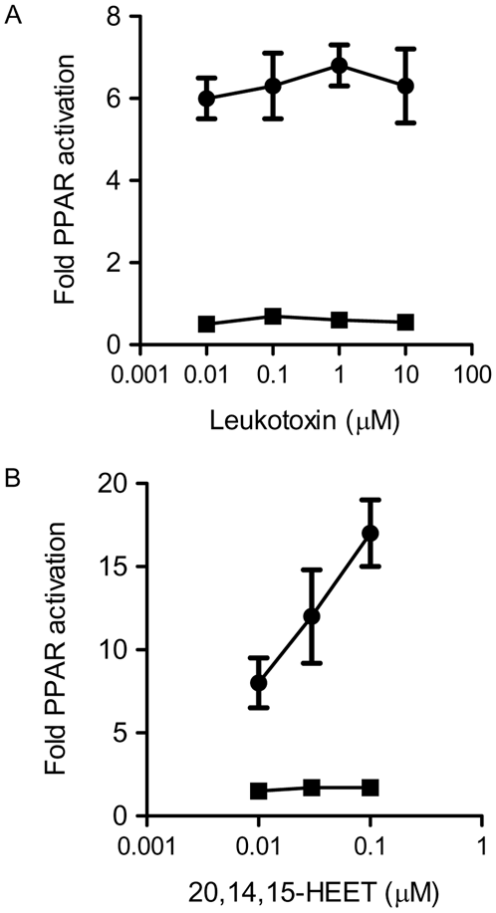
Alternative CYP products and PPARα activation. (A) PPAR reporter gene activation is not induced by the linoleic acid CYP2J2 metabolite leukotoxin in the presence or absence of PPARα, but is potently induced by (B) the CYP4A 14,15-EET metabolite 20,14,15-HEET in the presence of PPARα. HEK293 cells were transfected with PPAR reporter gene in the presence or absence of PPARα. 20 h post transfection cells were treated with CYP2J2 products (0–10 µM), for a further 20 h.

8,9- and 11,12-EET at nM concentrations induced activation of PPAR in HEK293 cells in the presence, but not absence of transfected PPARα (Figure 4). The CYP products 14,15-EET, or 5,6-DiHETE, the stable metabolite of 5,6-EET (Figure 4) or the linoleic acid metabolite of CYP2J2 leukotoxin (Figure 5A) had no effect on PPARα reporter gene activation. Although, 14,15-EET had no effect in our hands, consistent with the previous report [5], the CYP4A hydroxylase 14,15-EET metabolite was a potent PPARα activator (Figure 5B). The activation of PPARα responses by 8,9-EET or 11,12-EET was completely reversed when cells were co-transfected with dominant negative DN-PPARα (Figure 4B).

### PPARα activation induces PDK4 in cardiac tissue *in vitro*

**Figure 6:**
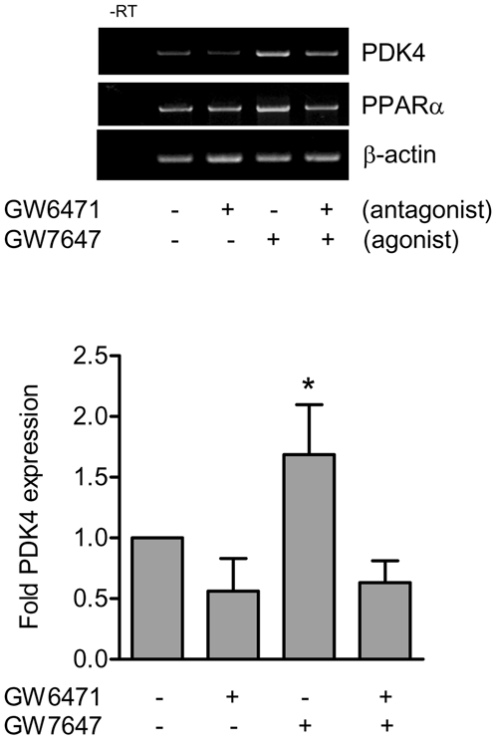
PPARα activation induces the PPARα target gene PDK4 in heart segments in organ culture. Fresh mouse heart tissue in culture was incubated in the presence or absence of the selective PPARα agonist GW7647 (10 nM) or antagonist GW6471 (3 µM) for 24 h and PDK4, PPARα and β-actin expression determined by RT-PCR. *denotes p<0.05 by one-sample t-test between control and treatments. Data represents mean±SEM of n = 9 incubations from 3 separate experiments.

PDK4 is a tissue specific PPARα target gene that facilitates fatty acid oxidation by “sparing” pyruvate for oxaloacetate formation [12], [13]. The highly selective PPARα ligand GW7647 induced PDK4 mRNA in mouse cardiac tissue in culture *in vitro* (Figure 6); an effect which was abolished by co-incubation with the selective PPARα antagonist GW6471 (Figure 6), or if tissue was used from PPARα knockout mice (data not shown).

### Cardiac-specific CYP2J2 transgenic mice have an elevated PPARα response during fasting

The fasting response is a model of PPARα activation *in vivo* as a decline in insulin levels and/or a rise in lipid fuel availability facilitates PPARα activation and the up-regulation of PDK4. Moreover, this marked up-regulation of PDK4 expression in response to fasting is absent in PPARα knockout mice [14]. Therefore, PDK4 is a robust index of PPARα activation *in vivo*. Expression of the related proteins PDK1, PDK2 and E1a are not regulated by PPARα, and were used as controls.

**Figure 7:**
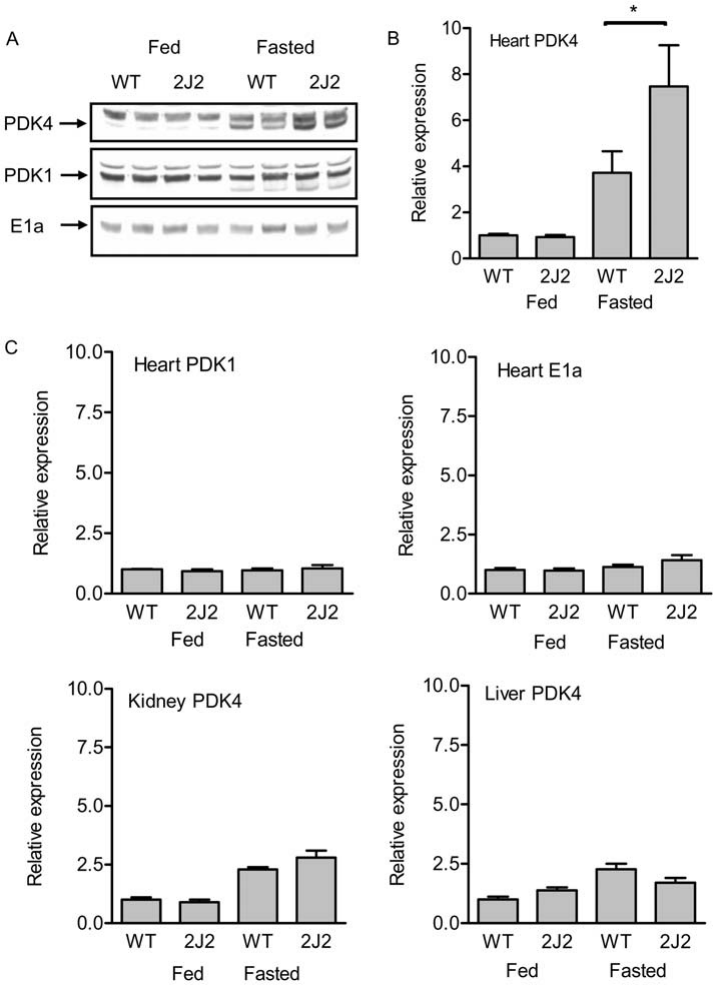
CYP2J2 augments PPARα *in vivo* in the fasting model of PPARα activation. Female wild-type or cardiac-specific CYP2J2 Tr mice were allowed food and water ad libitum, or fasted for 24 h. Figure (A) shows representative western blots for 2 of the 6 animals tested for PDK4, PDK1 (antibody has cross reactivity with PDK4 indicated by changes in the lower band) and E1a in the hearts of wild type (WT) or cardiac-specific CYP2J2 Tr mice (2J2); specific bands are identified by the arrows. Figures show the relative protein expression of PDK4 in the heart (B), kidney, and liver (C as indicated) and PDK1 and E1a in the heart (as indicated) in wild type (WT) and cardiac-specific CYP2J2 Tr (2J2), fed and fasted female mice. Data represents relative densitometry of protein compared to wild type fed controls for n = 4−6 separate animals in each group. Only the PPARα target gene PDK4 was induced on fasting both in the heart and kidney. Upon fasting there was an approximate doubling of PDK4 in the hearts (b), but not the kidney (e), or liver (f) of female cardiac specific CYP2J2 transgenic mice. * denotes p<0.05 by unpaired t-test between the fasting response in wild-type and CYP2J2 transgenic mice.

Cardiomyocyte-specific CYP2J2 transgenic (Tr) mice have been generated and have a normal heart anatomy and contractile function [15]. Fed CYP2J2 Tr mice had no altered expression of PDK1, -2, -4 or E1a expression in the heart, kidney, or liver compared to wild type controls (Figure 6; and data not shown). In fasted mice, PDK4 protein expression was selectively up-regulated in the heart (Figure 7A and B), kidney and liver (Figure 7C) of wild type mice. In response to fasting, wild type male mice had an approximate 2–3 fold higher induction of cardiac PDK4 expression than female mice (9.3±2.4 male compared to 3.7±0.9 female; relative fold induction; n = 4−6). The basal PDK4 levels between male and female mice were equivalent, so this gender difference in PPARα activity/PDK4 expression upon fasting is gender specific.

Upon fasting, male wild type and CYP2J2 Tr mice, had a comparable induction of cardiac PDK4 protein (9.3±2.4 wild type; 7.4±1.3 CYP2J2 Tr; fold expression; n = 4). In contrast, female CYP2J2 Tr exhibited a much greater induction of cardiac PDK4 protein upon fasting compared to wild- type controls (Figure 7). PDK1, PDK2 and E1a protein expression were unchanged by 24 h of fasting in any tissue tested (Figure 6, and data not shown).

Up-regulation of PDK4 expression is linked to a decline in circulating insulin concentrations [13], [14], [16]. Upon fasting, both plasma insulin and blood glucose levels fell to equivalent levels in wild type and CYP2J2 Tr mice (Table 1). Fibrate administration is associated with suppression of circulating triglyceride levels [2], however, neither triglyceride nor non-esterified fatty acid concentrations were affected in wild type or CYP2J2 Tr mice (Table 1). Since no systemic metabolic differences were observed, any changes in PPAR response we conclude are due to the local cardiac specific activity of CYP2J2 in the transgenic mouse.

**Table 1:** Blood parameters in fed and fasted wild type and cardiac-specific CYP2J2 transgenic mice (CYP2J2).

	Fed		Fasted	
	Wild Type	CYP2J2	Wild Type	CYP2J2
**Insulin (µU/ml)**	13±3	11±2	3±1*	3±1*
**Glucose (mM)**	13.3±0.3	12.5±0.6	4.5±0.6*	5.5±0.5*
**NEFA (mM)**	1.13±0.04	1.12±0.05	0.91±0.18	0.92±0.14
**Triglycerides (mM)**	0.7±0.1	0.9±0.1	0.5±0.2	0.7±0.1

Wild type and CYP2J2 mice have similar basal levels of plasma insulin, blood glucose, non-esterified fatty acids (NEFA) and triglycerides. Following 24 h of fasting, plasma insulin and blood glucose dropped in both wild type and CYP2J2 mice to equivalent levels, while non-esterified fatty acids and triglycerides remained relatively unchanged. This data represents the mean±s.e.m. for n = 6 animals per group. * denotes p<0.05 by unpaired t-test between fed and fasted levels.

### Endogenous CYPs and the cardiac fasting response

The use of pharmacological CYP inhibitors *in vivo* is complicated due both to lack of specificity of inhibitors and the great heterogeneity in CYP enzymes between species. We did however examine the fasting response in CYP2J5 knockout mice [17], the only murine CYP2J family member where a knockout has been generated. There was however no difference in the circulating blood glucose levels, or the heart, liver or kidney PDK4, or heart E1a expression levels (Table S1) between knockout and wild type male or female mice either under fed or fasted conditions.

## Discussion

The nature of endogenous PPAR ligands are still far from clear, as is whether PPARs act as general lipid sensors or whether high affinity ligands exist in the body. Here we show CYP2J2 can act as an endogenous epoxygenase source of high affinity PPAR ligands. When co-transfected together *in vitro*, CYP2J2 induces PPAR, in particular PPARα, activity. In cardiac-specific-CYP2J2 Tr mice, fasting greatly elevates the PPARα target gene PDK4. These results do not exclude a role for CYP2J2 or other CYPs as regulators of PPARβ/δ or –γ. Indeed we found CYP2J2 can activate PPARδ and PPARγ, (albeit it to lower absolute levels then PPARα in our transfection system) and it is known that lipid CYP products (though not the CYP responsible) are endogenous PPARγ activators, induced by laminar shear of human endothelial cells *in vitro* [7], [8].

Unlike other proposed PPAR ligand-generating enzymes (e.g. 12/15-lipoxygenase; [18]), CYP2J2 did not require additional arachidonic/linoleic acid substrate(s), suggesting a high level of functional coupling between the epoxygenases and PPARs. We also show for the first time a functional *in vivo* response for a PPAR ligand generating system. Our results do not rule out the role of other enzymes, such as phospholipases [3] or lipoprotein lipases [4] implicated in PPARα ligand generation. These enzymes are likely to produce PPAR ligands in parallel to CYPs, and/or supply free fatty acid substrates for CYPs to utilise.

8,9-EET, and 11,12-EET, but not 14,15-EET activated PPARα. 11,12-EET in contrast to 14-15-EET is highly anti-inflammatory and vascular protective [10], [19]. Therefore, we propose that PPARα is a likely anti-inflammatory target for 11,12-EET and CYP2J2. Indeed we found the combination of PPARα and CYP2J2 abolished IL-1β induced NFκB activation; a central pro-inflammatory transcription factor and PPARα target [1], [2].

Many EETs, including 14,15-EET, can also act as cellular hyperpolarising agents [9], [19], however, since 14,15-EET was inactive in our system, hyperpolarization mechanisms are highly unlikely to be involved. Our results are consistent with previous findings that EETs and some of their metabolites can directly bind and activate PPARα [5]–[7]. Although 14,15-EET did not activate PPARα in our hands, its CYP4A hydroxylase 14,15-EET metabolite 20,14,15-HEET, was the post potent EET product we tested. EETs can be rapidly metabolised by at least 10 different intracellular pathways, and it is estimated that when given exogenously <10% is available free within the cell [9]. Our results therefore do indicate that alternative CYP2J2 products exist or further unknown EET metabolites [5], [7], [8] are potential endogenous PPARα activators.

There is considerable species difference between CYPs in man and in the mouse. CYP2J2 is the human isoform, in the mouse the situation is far more complex with up to 8 putative homologues (CYP2J5 – CYP2J13; [20]). Since epoxygenases are ubiquitous and potentially have many roles, examining the role of endogenous epoxygenases especially in the mouse is extremely difficult. We therefore chose as our main model the established cardiac specific CYP2J2-Tr mouse. We did however test the recently described CYP2J5 knockout mouse [17], the only CYP2J knockout available. However, we did not detect a change in the fasting response or in PDK4 expression, suggesting a lack of involvement of CYP2J5 in PPARα ligand generation or the more likely compensation from other mouse CYP2J or CYP2C EET-producing epoxygenases that are present.

The selective augmentation of PDK4 in cardiac-specific CYP2J2-Tr mice occurred only in female mice. The fasting PPARα response was much stronger in males, and we believe maximally activated. Interestingly, our results are consistent with known gender differences in cardiac PPARα responses in the mouse. Pharmacological stress of the hearts of PPARα knockout mice with Etomoxir to prevent mitochondrial fatty acid import, results in cardiac lipid accumulation and a 100% mortality of male mice but only 25% mortality of female mice [21].

In conclusion, i*n vitro* CYP2J2 activates PPARα without exogenous stimuli. I*n vivo* CYP2J2 does not appear to be rate-limiting as PPARα target gene (PDK4) expression is only augmented in cardiac-specific CYP2J2 transgenic mouse upon fasting. Therefore, CYP2J2 *in vivo* is an enzyme apparently quiescent, but capable or responding to changes in lipid availability to generate endogenous PPARα agonists and thereby integrate transcriptional fasting events. CYP2J2 products activate PPARs, in particular PPARα *in vitro* and *in vivo*. As lipid-metabolising CYP enzymes have a widespread expression, utilise a variety of lipid substrates and produce a large family of oxidised biologically active lipid mediators, we suggest that lipid metabolising CYPs may represent an important source of PPAR ligands throughout the body.

PPARα is known as a controller of lipid metabolism and inflammation. Linking CYP2J2 and epoxygenases to PPARα has many potential clinical implications. Variants of CYP2J2 with lower activity are known in some populations to be linked to an increased risk of coronary artery disease [22], [23]. Epoxygenases such as CYP2J2 in addition to metabolising arachidonic acid may also regulate xenobiotic drug metabolism. Understanding how epoxygenases are regulated, the mediators they produce, and where they work, will give us novel information on biomarkers for dyslipidaemia and inflammation, allow us to understand side-effects of drugs metabolised by epoxygenases, and help us to design novel PPARα ligands based on the structure of high affinity EETs and their metabolites.

## Materials and Methods

### Materials

HEK293 cells were from ATCC. pEGFPN-1 and pNFkB-luc were from Clontech. pGL-2 was from Promega (Southampton, UK). pCMXmPPARα, pACOg.Luc, and h6/29 hPPARα were gifts from Dr Ruth Roberts (AstraZeneca; Maccelsfield, U.K.), pCMX-mPPARδ was from Dr Ronald Evans (Salk Institute, La Jolla, USA), pDR-1 was from Dr Bert Vogelstein (Johns Hopkins University, Baltimore, USA), pCMX-mPPARγ was from Dr Christopher Glass (UCSD, San Diego, USA). Novafector was from VennNova (Pompano Beach, FL, USA). Rabbit polyclonal anti-CYP2J2 [24] and PDK2 [25] were raised as previously described. Anti-PDK4 antibodies were generously provided by Professor Bob Harris (Indianapolis, USA). CYP2J2 metabolites were from Cayman Chemical Company (Axxora, Nottingham, UK). SKF525A was from Biomol (Affiniti Research Products, Exeter, UK). Plasma insulin ELISA was from Mercodia (Uppsala, Sweden). Plasma glucose kits were from Roche Diagnostics (Lewes, East Sussex, UK). WAKO kits for plasma triacylglycerol were from Alpha Labs. (Eastleigh, Hants, UK). ECL reagents, hyperfilm were from Amersham Biosciences (Little Chalfont, Buckinghamshire, UK). Bradford reagents for protein estimation were purchased from BioRad Ltd. (Hemel Hempstead, Hertfordshire. UK). All other reagents were from Sigma (Poole, Dorset, UK).

### Cell culture and transfections

HEK293 were maintained in DMEM containing, supplemented with Antibiotic/Antimycotic mix, and 10% FCS; 37°C; 5% CO_2_; 95% air. Cells were transfected with Novafector and Luciferase assays performed, essentially as previously described [26] but modified for a 96 well format [27]. Luciferase activity was normalised to cell protein (BCA assay). Global cellular changes, cell morphology, and GFP expression were recorded on a Nikon TE2000 inverted florescent microscope, with a SPOT RT digital camera. In some experiments organ culture of mouse cardiac tissue was performed, essentially as previously described [28].

### Ethics Statement

All animal studies were approved by the National Institute of Environmental Health Sciences Animal Care and Use Committee.

### Animal experiments

Cardiac-specific CYP2J2 transgenic mice (α−MHC promoter driven) and littermate wild type C57BL6/J controls [15] along with CYP2J5 knockout mice [27] have been described previously. Animals were allowed food and water ad libitum or fasted for 24 h. In some experiments mice were given SKF525A (30 mg/kg; i.p) or vehicle (sterile PBS) immediately prior to initiation of the 24 h fasting/non-fasting period.

### Immunoblotting and assays

PDK1, -2, -4 and E1a and CYP2J2 protein levels were determined as previously described [13], [24], [25]. For animal experiments each representative immunoblot presented are results from a single gel exposed for a uniform duration, and each lane represents a preparation from a different mouse. Plasma immunoreactive insulin concentrations were measured by ELISA, using rat insulin as a standard. Plasma glucose concentrations were determined by a glucose oxidase method. Plasma NEFA and TAG levels were determined spectrophotometrically using commercial kits.

## Supporting Information

Table S1 Blood glucose and PDK4 expression in female fed and fasted wild type and CYP2J5 knockout (−/−) mice. Wild type and CYP2J5 −/− mice have similar basal levels of plasma glucose. Following 24 h of fasting, blood glucose dropped in both wild type and CYP2J2 mice to equivalent levels, while the PPARalpha target gene PDK4 was induced to similar levels in the heart, liver and kidney. The non-PPARalpha target genes E1a and PDK2 (data not shown), were unaffected by fasting. Similar results were found in male mice. This data represents the mean±s.e.m. for n = 4 animals per group. * denotes p<0.05 by paired t-test between fed and fasted levels.
(0.03 MB DOC)
