# Supplementary material for: The Epoxygenases CYP2J2 Activates the Nuclear Receptor PPARα In Vitro and In Vivo
Source: PLoS One. 2009 Oct 12;4(10):e7421. doi: 10.1371/journal.pone.0007421 (PMC2756622; doi:10.1371/journal.pone.0007421)
Supplement: Table S1 — Blood glucose and PDK4 expression in female fed and fasted wild type and CYP2J5 knockout (−/−) mice. Wild type and CYP2J5 −/− mice have similar basal levels of plasma glucose. Following 24 h of fasting, blood glucose dropped in both wild type and CYP2J2 mice to equivalent levels, while the PPARalpha target gene PDK4 was induced to similar levels in the heart, liver and kidney. The non-PPARalpha target genes E1a and PDK2 (data not shown), were unaffected by fasting. Similar results were found in male mice. This data represents the mean±s.e.m. for n = 4 animals per group. * denotes p<0.05 by paired t-test between fed and fasted levels. (0.03 MB DOC) [file pone.0007421.s001.doc]

Supplemental Table 1

|  | **Fed** |  | | **Fasted** | |  |
| --- | --- | --- | --- | --- | --- | --- |
|  | **Wild Type** | | **CYP2J5 -/-** | **Wild Type** | **CYP2J5 -/-** | |
| **Glucose (mM)** | 10.1±0.7 | | 9.2±1.2 | 4.8±0.6* | 5.8±0.6* | |
|  |  | |  |  |  | |
| **Heart PDK4** | 1.0±1.1 | | 1.5±0.1 | 17.2±5.7* | 21.3±6.3* | |
| **Heart E1α** | 1.0±0.1 | | 1.3±0.1 | 1.0±0.1 | 1.0±0.1 | |
|  |  | |  |  |  | |
| **Liver PDK4** | 1.0±0.1 | | 1.3±0.1 | 1.7±0.1* | 2.0±0.1* | |
| **Kidney PDK4** | 1.0±0.2 | | 1.0±0.1 | 1.7±0.2* | 1.8±0.3* | |

Blood glucose and PDK4 expression in female fed and fasted wild type and CYP2J5 knockout (-/-) mice. Wild type and CYP2J5 -/- mice have similar basal levels of plasma glucose. Following 24h of fasting, blood glucose dropped in both wild type and CYP2J2 mice to equivalent levels, while the PPARα target gene PDK4 was induced to similar levels in the heart, liver and kidney. The non-PPARα target genes E1a and PDK2 (data not shown), were unaffected by fasting. Similar results were found in male mice. This data represents the mean ± s.e.m. for n=4 animals per group. * denotes p<0.05 by paired t-test between fed and fasted levels.
